# Riboflavin transporter: evidence of a role as entry receptor for chimpanzee endogenous retrovirus

**DOI:** 10.1093/ve/veaf031

**Published:** 2025-05-07

**Authors:** Loai AbuEed, Ariko Miyake, Nashon Wanjala, Didik Pramono, Dimas Abdillah, Masanori Imamura, Masayuki Shimojima, Joachim Denner, Junna Kawasaki, Kazuo Nishigaki

**Affiliations:** Laboratory of Molecular Immunology and Infectious Disease, Joint Graduate School of Veterinary Medicine, Yamaguchi University, 1677-1 Yoshida, Yamaguchi City, Yamaguchi 753-8515, Japan; Research Institute for Cell Design Medical Science, Yamaguchi University, 1677-1 Yoshida, Yamaguchi City, Yamaguchi 753-8515, Japan; Laboratory of Molecular Immunology and Infectious Disease, Joint Graduate School of Veterinary Medicine, Yamaguchi University, 1677-1 Yoshida, Yamaguchi City, Yamaguchi 753-8515, Japan; Research Institute for Cell Design Medical Science, Yamaguchi University, 1677-1 Yoshida, Yamaguchi City, Yamaguchi 753-8515, Japan; Laboratory of Molecular Immunology and Infectious Disease, Joint Graduate School of Veterinary Medicine, Yamaguchi University, 1677-1 Yoshida, Yamaguchi City, Yamaguchi 753-8515, Japan; Research Institute for Cell Design Medical Science, Yamaguchi University, 1677-1 Yoshida, Yamaguchi City, Yamaguchi 753-8515, Japan; Laboratory of Molecular Immunology and Infectious Disease, Joint Graduate School of Veterinary Medicine, Yamaguchi University, 1677-1 Yoshida, Yamaguchi City, Yamaguchi 753-8515, Japan; Research Institute for Cell Design Medical Science, Yamaguchi University, 1677-1 Yoshida, Yamaguchi City, Yamaguchi 753-8515, Japan; Laboratory of Molecular Immunology and Infectious Disease, Joint Graduate School of Veterinary Medicine, Yamaguchi University, 1677-1 Yoshida, Yamaguchi City, Yamaguchi 753-8515, Japan; Research Institute for Cell Design Medical Science, Yamaguchi University, 1677-1 Yoshida, Yamaguchi City, Yamaguchi 753-8515, Japan; Molecular Biology Section, Center for the Evolutionary Origins of Human Behavior, Kyoto University, 41-2 Kanrin, Inuyama City, Aichi 484-8506, Japan; Department of Medical Neuroscience, Graduate School of Medical Sciences, Kanazawa University, 13-1 Takaramachi, Kanazawa City, Ishikawa 920-8640, Japan; Sapiens Life Sciences, Evolution and Medicine Research Center, Kanazawa University, 13-1 Takaramachi, Kanazawa City, Ishikawa 920-8640, Japan; Special Pathogens Laboratory, Department of Virology I, National Institute of Infectious Diseases, Gakuen 4-7-1, Musashimurayama City, Tokyo 208-0011, Japan; Institute of Virology, Free University Berlin, Robert von Ostertag-Straße 7, 14163 Berlin, Germany; Graduate School of Medicine, Chiba University, 1-8-1 Inohana, Chuo-ku, Chiba City, Chiba 260-0856, Japan; Laboratory of Molecular Immunology and Infectious Disease, Joint Graduate School of Veterinary Medicine, Yamaguchi University, 1677-1 Yoshida, Yamaguchi City, Yamaguchi 753-8515, Japan; Research Institute for Cell Design Medical Science, Yamaguchi University, 1677-1 Yoshida, Yamaguchi City, Yamaguchi 753-8515, Japan

**Keywords:** endogenous retrovirus, paleovirology, riboflavin transporter, CERV1

## Abstract

Endogenous retroviruses (ERVs) are remnants of ancestral viral infections in germ cells that constitute a substantial proportion of the mammalian genome and are assumed to provide molecular fossil records of ancient infections. Analysis of these sequences may reveal the mechanisms of virus–host co-evolution, viral endogenization, and extinction. Chimpanzee endogenous retrovirus 1 (CERV1), a gamma retrovirus, is estimated to have circulated within primates for ~10 million years, although it is now apparently extinct. In this study, we aimed to gain an understanding of how the extinct CERV1 was transmitted and endogenized. On the basis of the identification of CERV1 fossils in the primate genome and using the expression-cloning method with the human cDNA library, we found that riboflavin transporter human SLC52A2 served as a receptor for CERV1 entry. The ectopic expression of human and chimpanzee SLC52A2 and its related SLC52A1 in heterogenic cells confers susceptibility to infection by CERV1 and porcine endogenous retrovirus (PERV). Virus interference experiments have shown that CERV1 inhibits infection by PERV and *vice versa*. This finding indicates that CERV1 and PERV belong to the same virus interference group. CERV1 shows infection in a wide range of human and primate cells. Notably, CERV1 infection is observed in human cell lines that express human SLC52A2 abundantly but hardly express human SLC52A1. Although CERV1 has been established to be present at high copy numbers in the great apes (*Pan troglodytes*, *Pan paniscus*, and *Gorilla gorilla*) and 15 Old World monkey species of the Cercopithecinae and Colobinae subfamilies, it is absent in humans and orangutans. CERV1 gene expression is observed in primates, including chimpanzees, suggesting that CERV1 has co-evolved with its hosts. Our results suggest that ERVs may have conferred resistance to viral infections in a convergent evolutionary manner. These findings are significant not only for advancing the field of paleovirology but also in terms of gaining an understanding of the potential risks of viral infection with respect to xenotransplantation, such as that from pigs to humans.

## Introduction

Retroviruses can be classified as exogenous (exRVs) and endogenous (ERVs) retroviruses based on their mode of transmission. exRVs are transmitted horizontally between hosts and can induce malignant disease in susceptible individuals, whereas ERVs are ancient retroviral sequences that infect germ cells and are transmitted vertically to the offspring of different vertebrate hosts (Coffin et al. 1997). In the latter case, this mode of transmission can lead to the accumulation of a substantial proportion of ERVs in the mammalian genome *via* repeated germline re-infections (Belshaw et al. 2005). Notably, ERVs are gradually attenuated or inactivated by mutations occurring during viral replication and *via* post-insertional mutation events occurring during host genome replication over time (Gifford and Tristem 2003). Hosts frequently regulate the gene expression of ERVs *via* epigenetic mechanisms, including CpG methylation of nucleotides or histone modifications, and it is assumed that these events ensure that ERVs do not pose a threat to the host (Kassiotis 2023). Moreover, some ERVs undergo a process of domestication within their host organisms, thereby gradually acquiring physiological functions (Johnson 2019). In this regard, it has been demonstrated that ERVs impede the circulation of exogenous retroviruses; e.g. ERV envelope (Env)-derived proteins that restrict retrovirus infection have been well documented in several species, including chickens, cats, and mice, as well as in humans (Robinson et al. 1981; Ikeda et al. 1985; Ito et al. 2013; Blanco-Melo et al. 2017; Frank et al. 2022; Pramono et al. 2024). Moreover, endogenous viral sequences represent a fossil record of past infections, thereby facilitating the study of paleovirulence (Emerman and Malik 2010), which seeks to determine how ancient, possibly extinct, retroviruses infected host species. With the exception of chimpanzee endogenous retrovirus 1 (CERV1)/*Pan troglodytes* endogenous retrovirus 1 and CERV2, all 40 ERV groups in chimpanzees are represented by lineal homologues in humans (Yohn et al. 2005; Polavarapu et al. 2006). However, whereas studies have shed some light on the CERV2 infection process (Soll et al. 2010; Miyake et al. 2022), the detailed mechanisms of CERV1 infection have yet to be sufficiently determined. Accordingly, in this study, we aimed to gain an understanding of how CERV1, an extinct strain of gamma retrovirus found in chimpanzees, was originally transmitted and endogenized. We found that CERV1 began to endogenize within primate germlines by utilizing riboflavin transporters as receptors. Furthermore, our findings provide evidence that ERVs may confer resistance to viral infections in a convergent evolutionary manner.

## Materials and methods

### Cell culture

The cells were cultured in high-glucose Dulbecco’s modified Eagle’s medium (DMEM; FUJIFILM Wako Pure Chemical Corporation, Osaka, Japan) supplemented with 10% foetal bovine serum. The cells were then incubated in a 5% CO_2_ incubator at 37°C. In this study, we used the following cell lines: HEK293T (human embryonic kidney transformed with SV40 large T antigen) kindly provided by Dr Mari Kannagi (Tokyo Medical and Dental University); MDTF (*Mus dunni* tail fibroblast) kindly provided by Dr Yoshinao Kubo (Nagasaki University); HeLa (human), Cos7 (African green monkey), and Vero (African green monkey) obtained from the Cell Resource Center for Biomedical Research, Institute of Development, Aging and Cancer, Tohoku University; HT1080 (human), Hs68 (human), NCMDF (normal cynomolgus monkey dermal fibroblasts), and PK15 (pig) obtained from the Japanese Collection of Research Bioresources (JCRB) cell bank; and Chimp-0363M (chimpanzee fibroblasts) kindly provided by Dr Masanori Imamura (Kyoto University) (Kitajima et al. 2020) and transformed with SV40 small T antigen (Chimp-0363M-T cells). HEK293T cells persistently infected with PERV-A/C (Denner et al. 2003) were provided by Dr Joachim Denner (Free University Berlin). Additionally, GPLac cells harboring murine leukemia virus (MLV) Gag-Pol expression vector and pMXs retroviral vector carrying LacZ (Miyake et al. 2016), and PLAT-GP cells (kindly provided by Dr. Toshio Kitamura, The University of Tokyo) harboring MLV Gag-Pol expression vector were cultured under the same conditions.

### Pseudo-typed virus preparation

Approximately 5 × 10^5^ GPLac cells were seeded in 6-well plates 1 day prior to transfection. The cells were transfected with 2.5 μg Env expression plasmid using TransIT®-293 reagent (Takara, Shiga, Japan) to produce LacZ-carrying Env-pseudotyped viruses. After 48 h, cell supernatants were collected, filtered through a 0.22-μm filter (Merck, Darmstadt, Germany), and stored at −80°C. The following Env expression plasmids were used for pseudo-typed virus preparations: pFUΔss CERV1 (CERV1 env) (Soll et al. 2010; Miyake et al. 2022), pFUΔss CERV1 Env-10346 [gene accession number, NC_072421.1 (position 34511146–34509476)], pFUΔss CERV1 Env-10346 L241P (substitution of proline at position 241 with leucine in the Env-10346 protein), pFUΔss PERV-A (PERV-A env) (Miyake et al. 2016), pFUΔss PERV-A/C (PERV-A 14/220 env) (accession number, GenBank: AAT77168.1), and pFUΔss 4070A (amphotropic MLV 4070A env) (Miyake et al. 2016). pFUΔss CERV1 Env-10346 and pFUΔss PERV-A/C (PERV-A 14/220 env) were constructed *via* gene synthesis and were Myc-tagged at the 3′ end (Eurofins Genomics K.K., Tokyo, Japan). pFUΔss CERV1 Env-10346 L241P was constructed *via* site-directed mutagenesis using the following primers: Fe-896S, 5′-GTACCAGTGGTGGGGCGGAGAGGAGCAGATTGATCCCAGA-3′, and Fe-920R, 5′-TCTGGGATCAATCTGCTCCTCTCCGCCCCACCACTGGTAC-3′. Mutagenesis was performed using the KOD-ONE Blue system (Toyobo, Osaka, Japan) in accordance with the manufacturer’s protocol. The resulting mutants were validated through sequencing.

### Infection assay

Cells were inoculated with the virus in 24-well plates containing 10 μg/ml polybrene (Nacalai Tesque, Inc., Kyoto, Japan) for 2 h. After the addition of fresh medium, the cells were cultured for 2 days post-infection. To conduct the LacZ assay, culture supernatants were discarded, and the cells were fixed with 250 μl of 2% glutaraldehyde for 15 min at room temperature and then stained with 250 μl of 5-bromo-4-chloro-3-indolyl-β-d-galactopyranoside (X-Gal) solution. After incubation at 37°C for 2 h, the nuclei in LacZ-positive cells were counted using an Olympus CKX31 microscope (Olympus, Tokyo, Japan), and images were captured using a camera.

### Viral interference assay

For the purpose of interference assays, HEK293T cells persistently infected with replication-competent PERV-A/C (Denner et al. 2003) and HEK293T cells seeded in 24-well plates were used as target cells. In addition, HEK293T cells were transfected with the CERV1 Env expression plasmid or empty vector (termed 293 T/CERV1 or 293 T/empty cells, respectively) using TransIT®-293 reagent (Takara), and as target cells, were thereafter cultured for 24 h prior to viral infection. Target cells were infected with each type of Env-pseudotyped virus in the presence of polybrene for 2 days and subsequently stained with X-Gal in accordance with the method described in the ‘Infection assay’ subsection. Single-cycle infectivity was assessed by counting the number of blue-stained nuclei.

### Receptor screening

CERV1 Env-pseudotyped virus has previously been shown to infect HEK293T cells (Miyake et al. 2022), and consequently, we screened a HEK293 cDNA library for genes encoding receptors potentially used by CERV1 for entry. A retroviral HEK293T cDNA library was constructed using a pMX retroviral vector. Poly(A)^+^ RNA was prepared from HEK293 cells using a FastTrack kit version 2.0 (Thermo Fisher Scientific, Waltham, MA, USA) according to the manufacturer’s protocol. cDNA was synthesized from the RNA using oligo dT_12–18_ primers, random hexamers, and Superscript II Reverse Transcriptase (Invitrogen, Carlsbad, CA, USA). Double-stranded cDNA was synthesized using DNA ligase, DNA polymerase I, and RNase H. Blunt-end cDNA was adapted using a *Bst*XI adaptor (Invitrogen) and ligated to the *Bst*XI sites of the pMX vector. The ligated DNA was then amplified in *Escherichia coli* DH10B competent cells, and plasmid DNA was extracted from cells using a Qiagen Plasmid Midi Kit (QIAGEN, Tokyo, Japan).

MDTF cells, which are resistant to CERV1 infection (Miyake et al. 2022), were transduced with the retroviral HEK293 cDNA library using a Retrovirus Packaging Kit Ampho (Takara, Kusatsu, Japan). PLAT-GP cells stably transduced with MLV Gag-Pol were transfected with CERV1 Env plasmid and either retroviral vector (pMSCV-puro) (Clontech, Mountain View, CA, USA) or pMXs encoding green fluorescent protein (GFP) using TransIT®-293 reagent (Takara). The supernatants were collected 2 days later and filtered (0.22 μm) to prepare viral suspensions, which were stored at −80°C until used for analysis. The viral titre was > 1 × 10^5^ IU/ml. The CERV1 Env-pseudotyped virus was also used to challenge MDTF cells transduced with the cDNA library in eight 10-cm-diameter culture dishes, with the cells being selected using 6 μg/ml puromycin (InvivoGen, San Diego, CA, USA). The puromycin-resistant colonies were plated onto culture plates, and these cells were subsequently challenged with CERV1-enveloped viruses carrying GFP. Sequence analysis revealed that the cDNAs encoded the riboflavin transporter human solute carrier 52A2 (huSLC52A2) in all GFP-positive colonies.

### Establishment of cell lines expressing SLC52A2 and SLC52A1

Human SLC52A2 cDNA was polymerase chain reaction (PCR)-amplified using cDNA from HEK293T cells as a template with KOD One Master Mix (Toyobo) and the primer pair huPAR1-F1 (5′-CTAGAATTCGCCACCATGGCAGCACCCACGCCCGCCCG-3′) and huPAR1-R1 (5′-GTGAAGATCTTCAAGCGTAATCTGGAACATCGTATGGGTAGGAGTCACAGGGGTCTGCACAGTCCTTTC-3′), with the resulting amplicons being inserted into a pMSCVneo expression vector (Clontech, Mountain View, CA, USA). Human solute carrier 52A1 (huSLC52A1) (accession number NP_001098047.1), chimpanzee SLC52A2 (accession number XP_016801823.1), and chimpanzee SLC52A1 (accession number XP_001164395.4) cDNAs were commercially synthesized by Eurofins Genomics K.K. (Tokyo, Japan). cDNA fused with a C-terminal hemagglutinin tag was also inserted into the pMSCVneo expression vector.

MDTF-chimpanzee SLC52A2 (MDTF-chSLC52A2), MDTF-human SLC52A2 (MDTF-huSLC52A2), and MDTF-human SLC52A1 (MDTF-huSLC52A1) cell lines were established. Briefly, PLAT-GP packaging cells expressing MLV Gag-Pol were co-transfected with expression vectors (pMSCVneo-chSLC52A2, pMSCVneo-chSLC52A1, pMSCVneo-huSLC52A2, or pMSCVneo-huSLC52A1) and a pFUΔss-Ampho 4070A MuLV Env expression vector using TransIT®-293 reagent, and then filtered (0.22 μm) supernatants were used to infect MDTF cells. Cells were cultured in DMEM containing 600 μg/ml neomycin.

### Detection of SLC52A1 and SLC52A2 expression by reverse transcription quantitative PCR

Total RNA was extracted from the cell lines HEK293T, HeLa, HT1080, HS68, NCMDF, Vero, Cos7, and chimpanzee cells using the RNeasy Mini kit (QIAGEN) in accordance with the manufacturer’s instructions. Thereafter, cDNA was synthesized using the PrimeScript II first-strand cDNA synthesis kit (Takara) according to the manufacturer’s instructions. Prior to reverse transcription, the RNA samples were treated with recombinant DNase I (Takara). The cDNA was amplified using SYBR Premix Ex Taq II (Tli RNase H Plus; Takara) in the CFX96 Touch real-time PCR detection system (Bio-Rad, Hercules, CA, USA). SLC52A2 was amplified using the primers Fe-995S (TTCCTGCTTACCCTACGGGC) and Fe-1012R (GGAAGCAGGCCAGGGGATT). SLC52A1 was amplified using the primers Fe-996S (TTGCTGTTGCCATCACTACC) and Fe-1013R (CAAAGCCTCTTCTTCCTCCTTC), and the internal control glyceraldehyde 3-phosphate dehydrogenase (GAPDH) was amplified using the primers hGAPDH-F-Tsu (5′-CACCACCATGGAGAAGGCTG-3′) and hGAPDH-R-Tsu (5′-GCTGATGATCTTGAGGCTGTTGT-3′). All reactions were performed using 8-well white strips (Bio-Rad Laboratories, Inc., Hercules, CA, USA) and run on a CFX96 Touch Real-Time PCR Detection System (Bio-Rad Laboratories, Inc., USA). Thermal cycling was performed according to the manufacturer’s instructions.

### Sequence analysis of SLC52A1 and SLC52A2 genes

PCR amplification was performed using the KOD One Master Mix (Toyobo) following the manufacturer’s instructions. cDNA from cultured cells—including HEK293T, HeLa, HT1080, HS68, NCMDF, Vero, Cos7, and Chimp-0363M cells—was used as the template. For SLC52A2 amplification, primers Fe-991S (5′-AGGTGGGAAAAGAACTGGCT-3′) and Fe-1010R (5′-CAGGGGCTTACCCTACAGC-3′) were used. For SLC52A1 amplification, primers Fe-993S (5′-GGGAAGGACCTGCCTGTGA-3′) and Fe-1011R (5′-GAGTGTGCAGGTGTCCATGA-3′) were used.

### Phylogenetic and sequence analyses

To identify CERV1 *env* gene sequences in primates, the CERV1 env amino acid sequence (Soll et al. 2010) was used as a query for a tBLASTn search against 41 primate genome databases (Supplementary Table 1) at the National Center for Biotechnology Information website (https://blast.ncbi.nlm.nih.gov/Blast.cgi, accessed 3 May 2024). The resulting sequences were filtered based on identity (≥80%) and sequence length (≥450 bp). All the identified CERV1 *env* genes are listed in Supplementary Table 2. Alignment was performed using Muscle (Edgar 2004), and MEGA 11 (Tamura et al. 2021) was used for phylogenetic analysis. A phylogenetic tree of the *env* genes was constructed based on the full-length open reading frame amino acid sequences (Supplementary Table 3) and generated using the neighbour-joining method (Saitou and Nei 1987) with the p-distance model (Nei and Kumar 2000). Phylogenetic trees for the SLC52A1 and SLC52A2 genes (Supplementary Table 4) were constructed using the maximum likelihood method (Edgar 2004). Substitution models were selected based on the lowest Bayesian information criterion score (JTT + G) (Jones et al. 1992). Bootstrap values from 1000 replicates were used as branch junction percentages to assess nodal support, and tree visualization was performed using iTOL (version 6.9.1) (Letunic and Bork 2024).

### Estimation of the integration timing of CERV1 in primates

An orthologue-dating method was employed to estimate the integration timing of CERV1 in primates (Aiewsakun and Katzourakis 2015). Initially, CERV1 *env* genes were identified using a tBLASTn search of the NCBI genomic database. Subsequently, 10-kbp sequences flanking the CERV1 *env* genes, both upstream and downstream, were extracted, and sequence similarity was analysed using BLASTN software. The presence or absence of orthologous *env* loci was assessed based on the alignment coverage of the flanking sequences, with the divergence times of the primate species being determined using the TimeTree database (Kumar et al. 2017).

### RNA-seq analysis

The expression levels of SLC52A1, SLC52A2, and CERV1 *env* were assessed using publicly available RNA-seq data obtained from the NCBI Sequence Read Archive (SRA) database (Leinonen et al. 2010). Data were pre-processed using fastp (version 0.20.1) (Chen et al. 2018) with specific parameters (−w 4, −y −3, and −x), and fasterq-dump (version 2.7.8a) was used to download the RNA-seq data. Genome index generation and mapping were performed using STAR (version 2.7.8a) (Dobin et al. 2013) with default settings. Expression levels were quantified as transcripts per million (TPM), and reads per kilobase of exon per million mapped reads were calculated using featureCounts (version 2.0.1) (Liao et al. 2014). The following tissues were not examined owing to the absence of RNA-seq data: placenta, colon, and lymph nodes in *Macaca mulatta*; and placenta, testes, and ovaries in *Macaca fascicularis* and *P. troglodytes*. The SRA run accessions, genome files, and gene annotation files are listed in Supplementary Table 5.

### Statistical analysis

Data are presented as the averages with standard deviations. The results of assays were considered statistically significant at *P* < .01, as determined using Student’s *t*-test.

### Ethics approval

Experiments were conducted according to the Guidelines for the Care and Use of Laboratory Animals of the Ministry of Education, Culture, Sports, Science, and Technology, Japan. All the experiments were approved by the Genetic Modification Safety Committee of Yamaguchi University.

## Results

### Identification of CERV1 entry receptors

It has been established that CERV1 Env-pseudotyped viruses can infect HEK293T cells (Miyake et al. 2022), and consequently, in the present study, we screened an HEK293 cDNA library for genes encoding receptors potentially used by CERV1 for entry. Given that MDTF cells are resistant to CERV1 infection (Miyake et al. 2022), MDTF cells transduced with the retroviral HEK293 cDNA library were challenged with CERV1-enveloped virus carrying a puromycin resistance gene, and 47 puromycin-resistant colonies were plated onto culture plates. To increase the specificity of the screening for receptor identification, these cells were subsequently challenged with CERV1-enveloped viruses carrying GFP, and among these 47 colonies, nine were identified as GFP-positive. Sequence analysis revealed that in all GFP-positive colonies, the cDNAs encoded the human riboflavin transporter (huSLC52A2).

**Figure 1:**
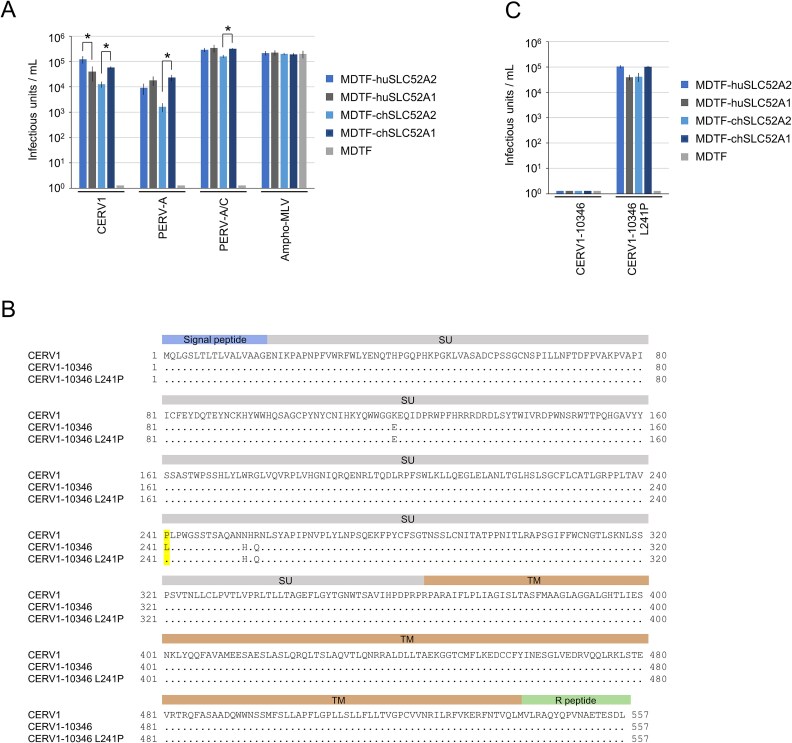
Infection of CERV1, PERV-A, and PERV-A/C. (A) Infection of LacZ-coding CERV1, PERV-A, and PERV-A/C Env-pseudotyped viruses in MDTF-huSLC52A2, MDTF-huSLC52A1, MDTF-chSLC52A2, MDTF-chSLC52A1, and MDTF cells. (B) Infection of LacZ-coding CERV1 10346 and CERV1-10346 L241P Env-pseudotyped viruses in MDTF-huSLC52A2, MDTF-huSLC52A1, MDTF-chSLC52A2, MDTF-chSLC52A1, and MDTF cells. (C) Alignment of the amino acid sequences of Envs of CERV1, CERV1-10346, and CERV1-10346 L241P. Envs are shown using single-letter amino acid codes. Leucine (L) and proline (P) at position 241 in CERV1 Env are indicated for comparison. Dots, conserved amino acid residues; SU, surface unit; TM, transmembrane domain. The data represent the averages of three independent experiments, with the standard deviations shown (A and B).

Having thus identified SLC52A2, we investigated whether this protein and the related SLC52A1 (huSLC52A1) protein (SLC52A2 and SLC52A1, respectively) function as CERV1 entry receptors. The amino acid sequence identity between SLC52A2 and SLC52A1 was 86.2% in *Homo sapiens* and 86% in *P. troglodytes* (Supplementary Fig. 1). SLC52A1 and SLC52A2 showed extremely high evolutionary similarity among humans and non-human primates (Supplementary Fig. 2). Chimpanzee SLC52A2-, human SLC52A2-, chimpanzee SLC52A1-, and human SLC52A1-expressing MDTF cells, designated MDTF-chSLC52A2, MDTF-huSLC52A2, MDTF-chSLC52A1, and MDTF-huSLC52A1, respectively, were characterized by permissiveness to CERV-1 Env-pseudotyped virus infection, whereas MDTF cells were not (Fig. 1A). These findings thus provided evidence to indicate that the expression of chimpanzee and human SLC52A2 and SLC52A1 in MDTF cells confers susceptibility to infection with CERV1-pseudotyped viruses. Furthermore, huSLC52A2 and huSLC52A1 have been established as entry receptors for porcine endogenous retrovirus subgroup A (PERV-A) and human tropic PERV-A/C (Ericsson et al. 2003). Viral infection using the PERV-A and PERV-A/C Env-pseudotyped viruses was confirmed in MDTF-chSLC52A2, MDTF-huSLC52A2, MDTF-chSLC52A1, and MDTF-huSLC52A1 cells (Fig. 1A), thereby indicating that chimpanzee and human SLC52A2 and SLC52A1 expression confers susceptibility to infection with pseudo-typed PERV.

To investigate the possibility that primates have functional CERV1 *env* genes in their genomes, we analysed publicly available whole-genome sequence data (Supplementary Table 1). Using the *env* gene of CERV1 as a reference (Soll et al. 2010), we downloaded all available CERV1 *env* genes from primate genomes (Supplementary Table 2) and extracted their full-length amino acid sequences (Supplementary Table 3). We then determined the full-length amino acid sequence with the highest similarity to the reference CERV1. The chimpanzee sequence Env-10346 [NC_072421.1 (position 34511146–34509476)] showed the highest similarity (99.28%) to the reference CERV1 (Fig. 1B). The CERV1 Env-pseudotyped virus using Env-10346 was assessed for infectivity; however, a loss of infectivity was detected. In the analysis of several amino acid substitutions in Env-10346 protein [substitution of glutamic acid at position 119 with lysine (E119K) and substitution of leucine at position 241 with proline (L141P)], infectivity was restored by substituting leucine at position 241 with proline in the Env protein (Fig. 1C), but not by E119K. This result shows that CERV1 *env* acquires infectivity through at least one amino acid change, but although 31 chimpanzee CERV1 *env* genes have been identified (Supplementary Table 3), it is currently unknown whether they retain their infectivity. Notably, a single amino acid change of Env in chimpanzees results in restoration of infectivity, suggesting that it has the potential to become a threat, although CERV1 has become extinct.

### Viral receptor interference of CERV1 and PERV-A

**Figure 2:**
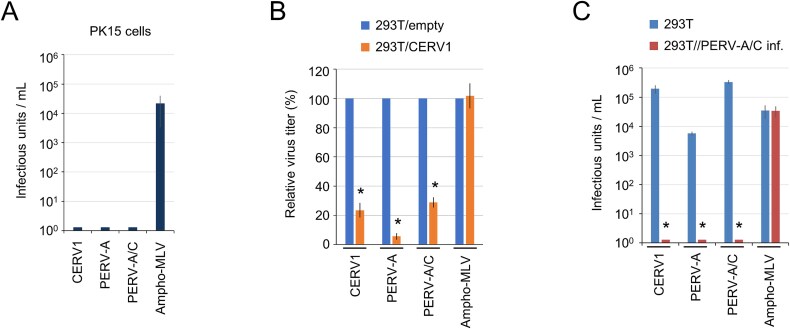
Interference group comparison of CERV1, PERV-A, and PERV-A/C. Infection of CERV1, PERV-A, and PERV-A/C Env-pseudotyped viruses in (A) PK15 cells, (B) HEK293T cells transiently expressing CERV1 Env (293/CERV1 cells) or empty plasmid (293/empty cells), and (C) HEK293T cells persistently infected with PERV-A/C (293T//PERV-A/C inf. cells) or 293T cells. Comparisons with HEK293T/empty or control cells were performed using Student’s *t*-test (^*^P < .01) in (B, C). The y-axis indicates infectious units per millilitre of virus (A, C) or relative virus titre (%) (B). The infectious titres for 293T/empty cells (B) are as follows. CERV1: (1.38 ± 0.62) × 10^5^ IU/ml PERV-A: (8.64 ± 4.36) × 10^3^ IU/ml, PERV-A/C: (2.09 ± 0.12) × 10^5^ IU/ml, Ampho-MLV: (9.16 ± 0.52) × 10^4^ IU/ml. The data represent the averages of three independent experiments, with the standard deviations shown.

The PK15 porcine kidney cell line, which produces PERVs, was found to be characterized by resistance to CERV1 infection, as well as infection with both PERV-A and PERV-A/C, by using pseudo-typed viruses (Fig. 2A). Therefore, we performed viral interference assays to determine whether CERV1 and PERV can be classified within the same interference group. We accordingly observed that infection with CERV1, PERV-A, and PERV-A/C Env-pseudotyped viruses was inhibited in the presence of CERV1 Env (Fig. 2B). Subsequently, HEK293T cells persistently infected with PERV-A/C (293T//PERV-A/C inf. cells) were assessed for their susceptibility to CERV1 infection and were found to be resistant to CERV1, PERV-A, and PERV-A/C infections (Fig. 2C), thereby indicating that CERV1 interferes with the infection of both PERV-A and PERV-A/C *via* at least Env-mediated interference. These results indicate that CERV1, PERV-A, and PERV-A/C belong to the same interference group.

### CERV1 infectivity in human and primate cell lines

**Figure 3:**
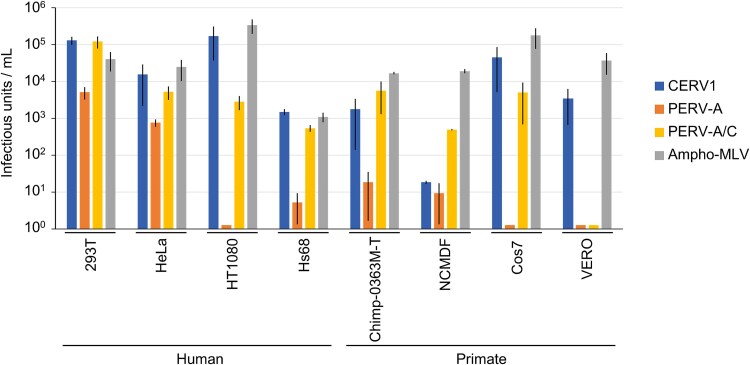
Comparison of the infectivity of CERV1, PERV-A, and PERV-A/C in human and primate cell lines. The infectivity of CERV1, PERV-A, and PERV-A/C Env-pseudotyped viruses was assessed in human and primate cell lines. The data represent the averages of three independent experiments, with the standard deviations shown.

As CERV1, PERV-A, and PERV-A/C utilize the same receptor, human and primate cell lines were assessed for susceptibility by Env-pseudotyped viruses (Fig. 3). CERV1 was found to infect humans (293T, HeLa, HT1080, and Hs68 cells), chimpanzees (Chimp-0363M cells), and African green monkeys (Cos7 and Vero cells), although not NCMDF. Contrastingly, PERV-A was found to infect HEK293T and HeLa cells but not other lines.

Moreover, with the exception of Vero cells, PERV-A/C was established to show infectivity in the human and primate cell lines assessed in this study. These findings indicate that despite using the same viral receptor, there are differences in susceptibility with respect to viral cell entry.

### Expression of SLC52A1 and SLC52A2 in human and primate cell lines

**Figure 4:**
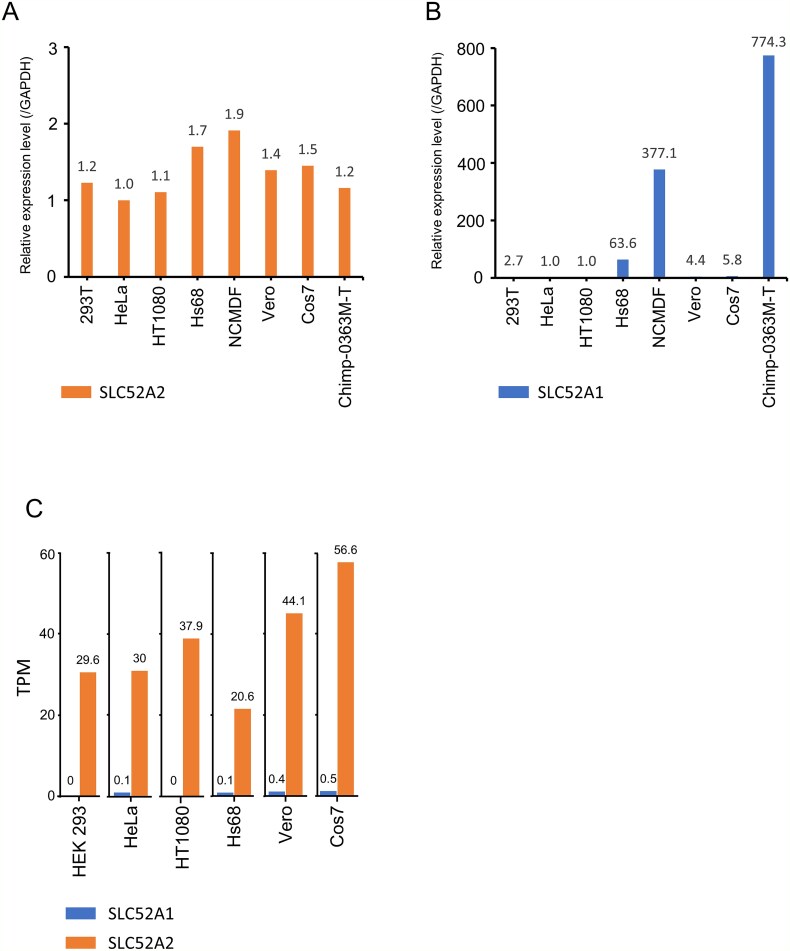
Expression of SLC52A1 and SLC52A2 in human and non-human primate cell lines. Quantification of SLC52A2 (A) and SLC52A1 (B) transcripts by quantitative RT-PCR in cell lines. The x-axis indicates the analysed samples. The y-axis indicates the expression level normalized to the expression of GAPDH. In each experiment, the normalized expression in HeLa cells is set to 1 for comparison across cell lines. (C) Expression of SLC52A1 and SLC52A2 in cell line. The x-axis indicates the analysed samples. The y-axis indicates TPM.

SLC52A1 and SLC52A2 mRNA expression was evaluated by reverse transcription quantitative (RT-q)PCR in total RNA extracted from various human and non-human primate cell lines (Fig. 4A and B). SLC52A2 expression was observed in all non-human primates (*P. troglodytes*, *M. fascicularis*, and *Chlorocebus sabaeus*) and human cell lines tested. In contrast, SLC52A1 expression was very low or undetectable in the 293T, HeLa, HT1080, Vero, and Cos7 cells, but high in chimp-0363M, NCMDF, and Hs68 cells. Furthermore, the gene expression levels of SLC52A1 and SLC52A2 in Hs68, Vero, and Cos7 cells were analysed using publicly available RNA-sequencing data (Supplementary Table 5). The expression data for SLC52A1 and SLC52A2 in HEK293, HeLa, and HT1080 cells were obtained from the Human Protein Atlas Database (Jin et al. 2023) (accessed 3 March 2025). The expression level of SLC52A1 was 0 or close to 0 in all cells tested in this study, while SLC52A2 was expressed abundantly (Fig. 4C). The results are consistent with those obtained by RT-qPCR. These results show that SLC52A2 is highly expressed across cell lines, whereas the expression level of SLC52A1 varies from highly expressed to barely expressed.

### Sequence analysis of SLC52A1 and SLC52A2 in human and primate cell lines

We analysed the sequences of SLC52A1 and SLC52A2 in the cell lines used in this study. The amino acid sequences of SLC52A2 were identical to the reference genome in all cases (Supplementary Fig. 3). As a characteristic amino acid, the 109th amino acid of SLC52A2 was serine in NCMDF cells derived from crab-eating macaques, but not in other cells. In contrast, SLC52A1 showed several substitution mutations across different cell lines (Supplementary Fig. 4). In the chimpanzee cell line, SLC52A1 showed the substitution mutation M200L; in the NCMDF cell line, the F181S mutation was observed; and in the African green monkey cell line, an L73S substitution mutation was observed (Supplementary Fig. 4). In the Hs68 cell line, SLC52A1 showed two substitution mutations, Q71R and A271V. In contrast, in HT1080, 293T, and HeLa cell lines, the nucleotide sequences of SLC52A1 were not determined due to the lower or undetectable expression by using nested PCR.

### Expression of SLC52A1 and SLC52A2 in human and non-human primate tissues

**Figure 5:**
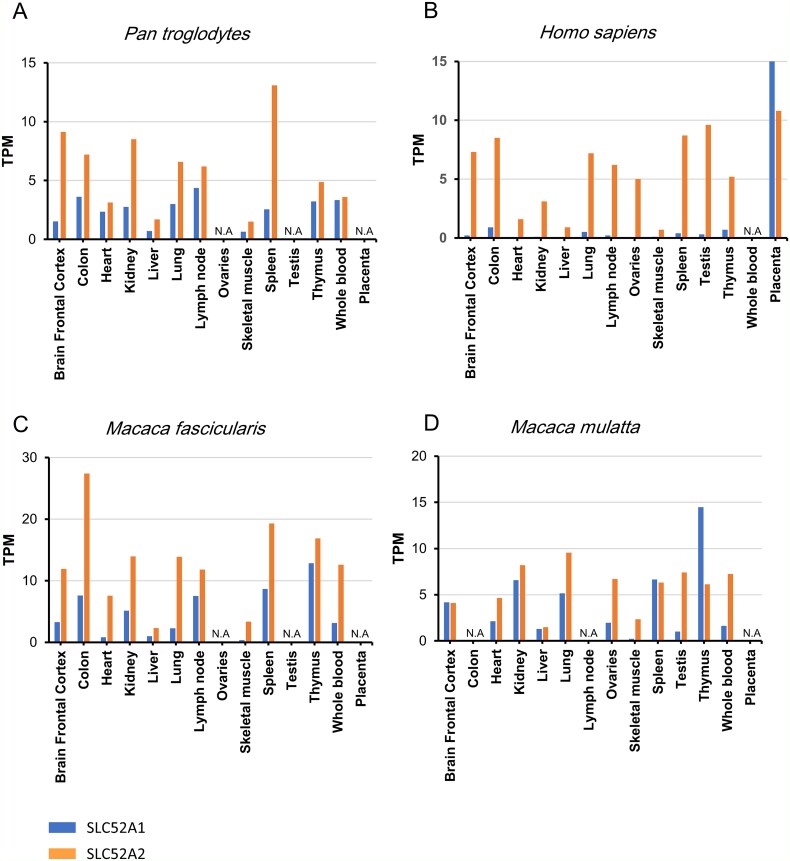
Expression of SLC52A1 and SLC52A2 genes in human and non-human primate tissues. (A–D) Bar graphs showing the expression levels of the SLC52A1 and SLC52A2 genes. The x-axis represents the tissues investigated, and the y-axis shows the normalized expression levels of SLC52A1 and SLC52A2 in TPM. N.A. indicates that expression levels in the specified tissue were not investigated due to the unavailability of RNA-seq data.

The gene expression levels of SLC52A1 and SLC52A2 in non-human primates were analysed using publicly available RNA-sequencing data from *P. troglodytes*, *M. fascicularis*, and *M. mulatta* (Supplementary Table 5). For humans, expression data were obtained from the Human Protein Atlas (Uhlén et al. 2015) (accessed 15 December 2024). Overall, SLC52A2 exhibited higher expression than SLC52A1 in most tissues across all species, with particularly low levels in the liver and skeletal muscle (Fig. 5A–D). In contrast, the expression levels of SLC52A1 were apparently different between human and non-human primates. In humans, SLC52A1 was highly expressed in the placenta, but expression levels were very low in other tissues (Fig. 5B). In non-human primates, SLC52A1 was expressed in all tissues and highly expressed in the thymus (Fig. 5B–D).

### Genetic diversity of CERV1 *env* in primates

A search of publicly available whole-genome data obtained from humans and primates revealed CERV1 *env* genes in great apes (*P. troglodytes, Pan paniscus,* and *Gorilla gorilla*) and 15 Old World monkey species from the Cercopithecinae and Colobinae subfamilies (Supplementary Table 1).

**Figure 6:**
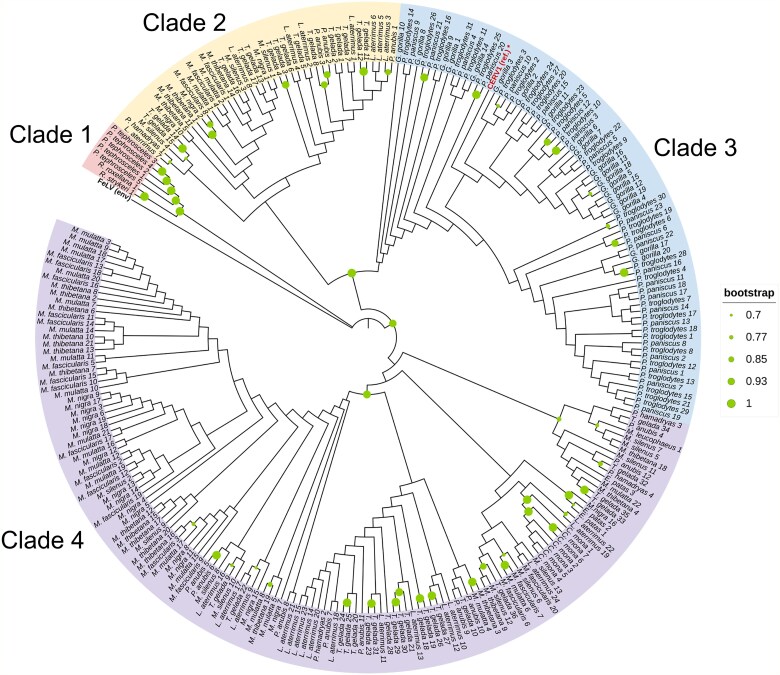
Phylogenetic analysis using the CERV1 Env amino acid sequences. The phylogenetic tree for the CERV1 Env amino acid sequences was constructed based on the neighbour-joining method. Bootstrap values >70% are shown adjacent to the respective branches. The tree is drawn to scale, with branch lengths representing the number of substitutions per site. The CERV1 env type is categorized as Clade 1, Clade 2, Clade 3, and Clade 4. The asterisk indicates the CERV1 Env reference sequence used in this study (Soll et al. 2010).

Contrastingly, CERV1 was not detected in the genomes of either humans or orangutans (*Pongo*), and similarly, *Nomascus leucogenys* and *Symphalangus syndactylus*, in the family Hylobatidae, do not harbour CERV1. The phylogenetic trees constructed based on env amino acid sequences revealed four distinct genetic clusters (Clades 1, 2, 3, and 4) within the CERV1 *env* sequences (Fig. 6), three of which (Clades 1, 2, and 4) were found in Old World monkeys, whereas Clade 3 was found exclusively in great apes. Furthermore, our phylogenetic analysis revealed that CERV1 *env* sequences from Old World monkeys and apes do not mix, which would tend to indicate an absence of any recent cross-species transmission of CERV1 between these species. However, we found that the copy number of the CERV1 *env* gene in the primate genome varied widely, ranging from 35 to 231 (Supplementary Table 2).

**Figure 7:**
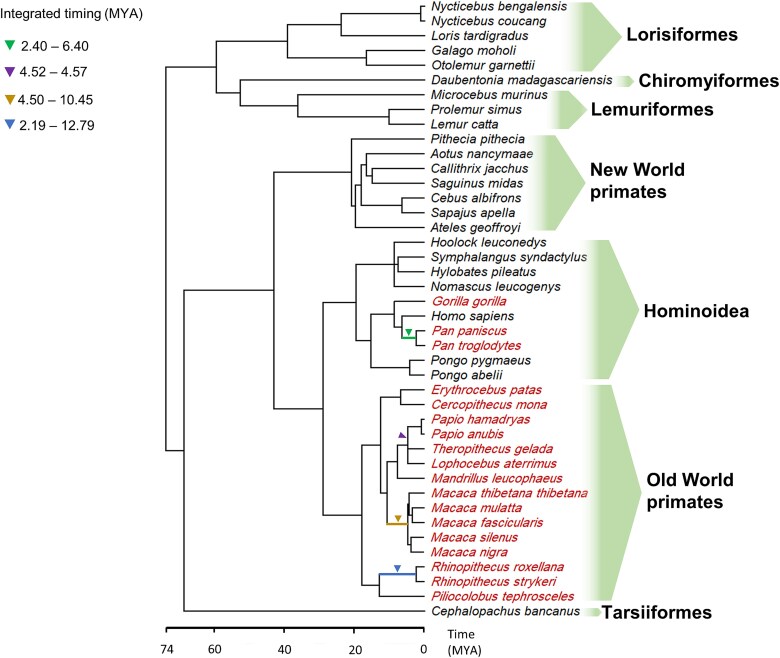
Evolutionary history of primate species with the integration timings of CERV1. The arrowheads indicate integration timings of the ancestor with CERV1. The red colour indicates the species infected with CERV1.

The timing of CERV1-related virus integration, which we determined based on an analysis of orthologous *env* genes in sister species, revealed that CERV1 probably emerged subsequent to a significant divergence between ape and Old World monkey lineages, as certain ape species were found to lack CERV1 (Fig. 7, Supplementary Table 6). In contrast, ancestral Old World monkeys were infected with CERV1 and transmitted the infection within their lineages several million years ago. Notably, the findings of our TimeTree phylogenetic tree analysis indicate that Old World monkeys may have been infected with CERV1 prior to apes.

### CERV1 *env* expression in tissues

**Figure 8:**
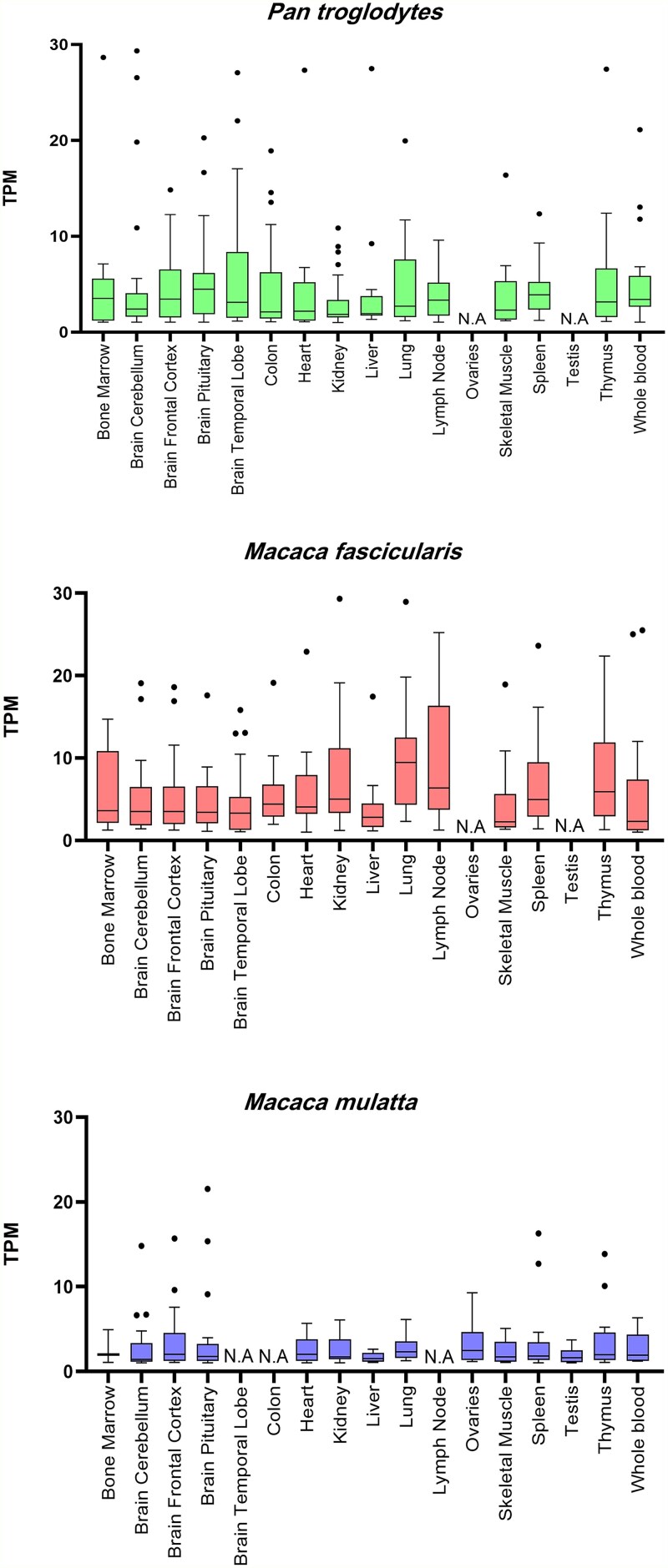
Expression of CERV1 *Env* in primate tissues. Box plot showing the CERV1 *Env* gene expression levels, analysed using publicly available RNA-seq data from *P. troglodytes*, *M. fascicularis*, and *M. mulatta*. Box-and-whisker plots depict the median as a line in the centre of a box bounded by the first and third quartiles, with whiskers to 1.5 times the interquartile range and outlier values shown as individual dots.

Tissue-specific expression of the CERV1 *env* gene was examined in *P. troglodytes*, *M. fascicularis*, and *M. mulatta*. CERV1 *env* expression was observed in most organs, with the highest levels of expression being detected in the lungs of chimpanzees, the thymus gland in *M. fascicularis*, and the brain frontal cortex in *M. mulatta* (Fig. 8). Comparatively, we detected low levels of CERV1 *env* expression in the livers of all three species. These results indicate that CERV1 may have co-evolved with the host subsequent to its endogenization.

## Discussion

Our findings in the present study provide evidence to indicate that CERV1 began to endogenize in primate germlines by utilizing riboflavin transporters as receptors ~10 million years ago. Riboflavin transporters have been identified as receptors of both PERV and modified retroviruses (Ericsson et al. 2003; Mazari et al. 2009), thereby indicating that the interaction between the retroviral envelope and the entry receptor is convergent in nature.

In the present study, CERV1 was found to be a viral interference group of PERV-A and PERV-A/C. This conclusion is based on the results showing the resistance of CERV1 infection to HEK293T cells persistently infected with PERV-A/C (Fig. 2C). Furthermore, it was suggested that this interference mechanism is mediated by the Env protein (Fig. 2B and C). These results suggest that the receptor for CERV1 is SLC52A1/2. PERV infection has been reported to have a greater function for SLC52A1 as an entry receptor (Ericsson et al. 2003; Marcucci et al. 2009). Non-permissive MDTF cells expressing chimpanzee and human SLC52A1/2 cDNA are highly susceptible to CERV1 infection. The infection with a pseudo-virus showed that MDTF cells expressing human SLC52A2 and chimpanzee SLC52A1 had a higher infection titre (Fig. 1A). Species-specific preferences regarding the usage of receptors may exist. However, the amount of receptor expression on the cell surface has not been measured, so at present, the preference for SLC52A1/2 is unknown for CERV1 infections. However, we do not intend to overemphasize this functional difference, but rather to show that there is a consistent significant difference in the experiments. Phylogenetic analysis of SLC52A1/2 showed high sequence similarity between species, suggesting that it is a riboflavin transport protein (Supplementary Fig. 2).

Analysis of the expression levels of SLC52A1/2 in human and non-human primate cell lines used in the present study showed that the expression levels of SLC52A2 were similar among them (Fig. 4A–C). The expression levels of SLC52A2 were considerably higher than those of SLC52A1 in all cells, while the expression levels of SLC52A1 varied from undetectable to high in cell lines. In particular, HEK293T, HeLa, and HT1080 cells exhibited extremely low or undetectable levels of SLC52A1. Interestingly, it has been reported that the inactive form of human SLC52A1 cDNA is expressed in cell lines and tissues. This is due to a 118 bp insertion between exon 2 and exon 3, inserting a stop codon due to a frameshift, and therefore, the cDNA encodes a 167 amino acid protein (Yonezawa et al. 2008). Therefore, SLC52A1 monitored by RT-qPCR is thought to be the sum of the normal form and inactive form, suggesting that the normal form of SLC52A1 was barely expressed (Fig. 4B and C). Indeed, we failed to determine the sequence of SLC52A1 in these cells. Notably, these cells are permissive for infection with CERV1 and PERV-A/C. SLC52A2 is expressed at extremely high levels in these cultured cells, so it is possible that viral infection occurs using SLC52A2 in these cell lines. Alternatively, even if SLC52A1 is expressed in relatively small amounts, it may function as a receptor for viral infection.

The results of the infectivity of PERV in cell lines in the present study are generally consistent with the results of previous studies (Takeuchi et al. 1998; Ericsson et al. 2003). The differences in infectivity between PERV-A and PERV-A/C in cell lines are due to regions outside the receptor binding region within Env, and this enhanced the infectivity of PERV-A/C in cells by stabilization of the envelope glycoprotein and increased receptor binding (Harrison et al. 2004).

It has been reported that the resistance of PERV to infection is related to the expression level and glycosylation of viral receptors (e.g. Vero and Cos7 cells) (Mattiuzzo and Takeuchi 2010). Furthermore, in rhesus macaques, crab-eating macaques, and baboons, SLC52A2 is genetically deficient for PERV infection due to the substitution of serine with leucine at amino acid position 109 in SLC52A2 (Mattiuzzo et al. 2007; Marcucci et al. 2009; Mattiuzzo and Takeuchi 2010).

In the present study, NCMDF cells were the only cells that did not become infected with CERV1. NCMDF cell are normal dermal fibroblasts and are classified as primary cultured cells that have not been immortalized. Both SLC52A1 and SLC52A2 are highly expressed (Fig. 4A and B), but the reason for the resistance to CERV1 infection is unknown. Since it was confirmed that NCMDF cells have a serine at position 109 of SLC52A2, it is thought that SLC52A1 functions as the entry receptor for PERV-A/C infection in NCMDF cells. On the other hand, it is unknown whether a serine at position 109 of SLC52A2 affects CERV1 infection.

As a result of experiments on the virus infection in various cell lines (Fig. 3), it was found that there was a difference in infectivity between CERV1 and PERV, even though they belong to the same viral interference group, suggesting different properties of the virus-receptor interaction.

In the present study, the expression analysis of SLC52A1/2 was only based on quantitative analysis of RNA, so the evaluation of the amount and localization of these endogenous proteins is a future issue.

The utilization of transport proteins for cell entry is a common feature of gammaretroviruses. For example, ecotropic MuLV utilizes mCAT1, the cationic amino acid transporter (Albritton et al. 1989). To date, all known receptors for feline and murine are multi-transmembrane receptors (Kozak 2014; Chiu et al. 2018). The discovery of SLC52A1/2 as an entry receptor for CERV1 follows this pattern of multipass membrane transport molecules acting as retroviral receptors (Yonezawa and Inui 2013). Some gammaretroviruses are known to share a receptor, such as GaLV, FeLV-B, KoRV-A, and 10A1-MuLV, which all use Pit1 (O'Hara et al. 1990; Johann et al. 1992; Takeuchi et al. 1992; Miller and Wolgamot 1997; Anderson et al. 2001; Oliveira et al. 2006); FeLV-D, ERV-DC, primate ERVs, and CERV2, which use CTR1 (Soll et al. 2010; Miyake et al. 2022; Tury et al. 2022); or FeLV-E and murine ERV, which use RFC1 (Miyake et al. 2019; Tsang et al. 2019). As shown in these examples, the interactions between retrovirus envelopes and entry receptors can be shown to be convergent in nature. Similar convergent evolution has been reported for antiviral molecules (e.g. Refrex-1) derived from the retroviral envelope (Miyake et al. 2022). It is extremely rare for a gammaretrovirus to utilize multiple entry receptors. There have been reports of FeLV-C mutant strains using molecules, such as THTR1, FLVCR1, and FLVCR2 (Shalev et al. 2009). In addition, although the receptor for FeLV-B is feline Pit1, some FeLV-B mutants have been found to use feline Pit2 (Anderson et al. 2001; Pramono et al. 2024), although their viral infectivity is low. On the other hand, CERV1, PERV, and CP virus (modified retrovirus) utilize both SLC52A1 and SLC52A2. One reason for this may be that the identities of SLC52A1 and SLC52A2 are very high (Supplementary Fig. 1). In the present study, the lack of evaluation of interactions of viral envelopes with endogenous receptors in the experimental system for identifying receptors is an issue for future research.

No remains of CERV1 infection are found in the human genome (Soll et al. 2010). This research has shown that CERV1 can infect human cell lines that express SLC52A2, regardless of the expression level of SLC52A1, suggesting that species-specific envelope-receptor incompatibility does not account for the absence of endogenous CERV1 copies in the human genome. Furthermore, SLC52A2 is widely expressed in human tissues (Fig. 5B), including the testes and ovaries, and SLC52A1 is highly expressed in the placenta, suggesting that the lack of endogenization is unlikely to result from the absence of receptor expression in the germline.

There are clear differences in the tissue expression of SLC52A1 between humans and non-human primates. In humans, the expression of SLC52A1 is limited to the placenta; in contrast, it is expressed in a wide range of tissues in non-human primates. This observation may be hypothesized to be due to the different roles of SLC52A1 in different species. Furthermore, the reason why CERV1 is not endogenized in humans may be related to the expression pattern of SLC52A1/2.

It is not yet clear why humans, orangutans, and New World monkeys do not possess endogenous CERV1. In addition to the pattern of receptor expression, factors such as the time and place of CERV1 prevalence may also be involved. Furthermore, the lack of CERV1 may be due to evolutionary resistance to viral infections, such as APOBEC3 or TRIM5α (Kaiser et al. 2007; Perez-Caballero et al. 2008). Furthermore, the results of the present study do not clarify the relationship between SLC52A1/2 gene polymorphisms and susceptibility to viral infection. However, it is known that there are more than 30 and 6 polymorphisms at the amino acid level in human SLC52A1 and SLC52A2, respectively, and the evolution of these genes may be expected. It is possible that there have been polymorphisms that confer resistance to CERV1 infection (Yonezawa and Inui 2013), but all of these are speculations, and this is a limitation of the current research.

Expression analysis of CERV1 in primates and the discovery of a viral receptor provide important insights into xenotransplantation from pigs to humans. Experimental primate models of pig organ transplantation have shown potential resistance to PERV infection (Specke et al. 2002, 2009). This resistance may be due to the fact that the SLC52A2 gene encodes serine at amino acid position 109 in macaque and baboon species, but this substitution is not present in humans, chimpanzees, or African green monkeys. At least, this mutation of serine may explain the resistance to PERV infection. Notably, it is not known whether this amino acid mutation in SLC52A2 affects the infectivity of CERV1. However, considering the timing of CERV1 endogenization in non-human primates, the 109 Ser of SLC52A2 is thought to have occurred after CERV1 endogenization. The mechanism of CERV1 endogenization may not involve the genetic change in SLC52A2. In the present study, the finding that CERV1 and PERV-A belong to the same interference group and the fact that the CERV1 *env* gene is expressed in primates suggest that it is possible that primates exhibit resistance to PERV infection. At present, it is not yet clear whether the endogenization of CERV1 confers resistance to PERV infection. However, the physiological significance of endogenized CERV2-related sequences as a defence against feline and primate retroviral infection has been reported (Miyake et al. 2022). Therefore, these gaps need to be tackled in future research.

Finally, since CERV1 belongs to the same interference group as PERV, the riboflavin transporter is the entry receptor for CERV1. However, it should be noted that the identification of the receptor is based on the results of experiments of ectopic expression of the receptors; it will be necessary to clarify this issue in the future using other approaches, such as receptor knockout experiments in cells.

## Conclusion

Collectively, the findings in this study will contribute to enhancing our understanding of the transmission trajectories of ancient viruses, provide clues as to why these viruses have not been endogenized in humans, and offer insights for research on human and primate evolution. In particular, given that ancient retroviruses have integrated into the genomes of humans and animals, the question of how these viruses infected their hosts represents a fundamental issue in the field of life sciences, which ideally needs resolution.

## Supplementary Material

FigureS1_veaf031

FigureS2_veaf031

FigureS3_veaf031

FigureS4_veaf031

Supplementary_Table_1-6_Virus_Evolution_veaf031

## Data Availability

The data presented in this study are openly available at https://github.com/loaiabueed/CERV1-receptor.
