# Supplementary figures and images for: Riboflavin transporter: evidence of a role as entry receptor for chimpanzee endogenous retrovirus
Source: Virus Evol. 2025 May 7;11(1):veaf031. doi: 10.1093/ve/veaf031 (PMC12202039; doi:10.1093/ve/veaf031)

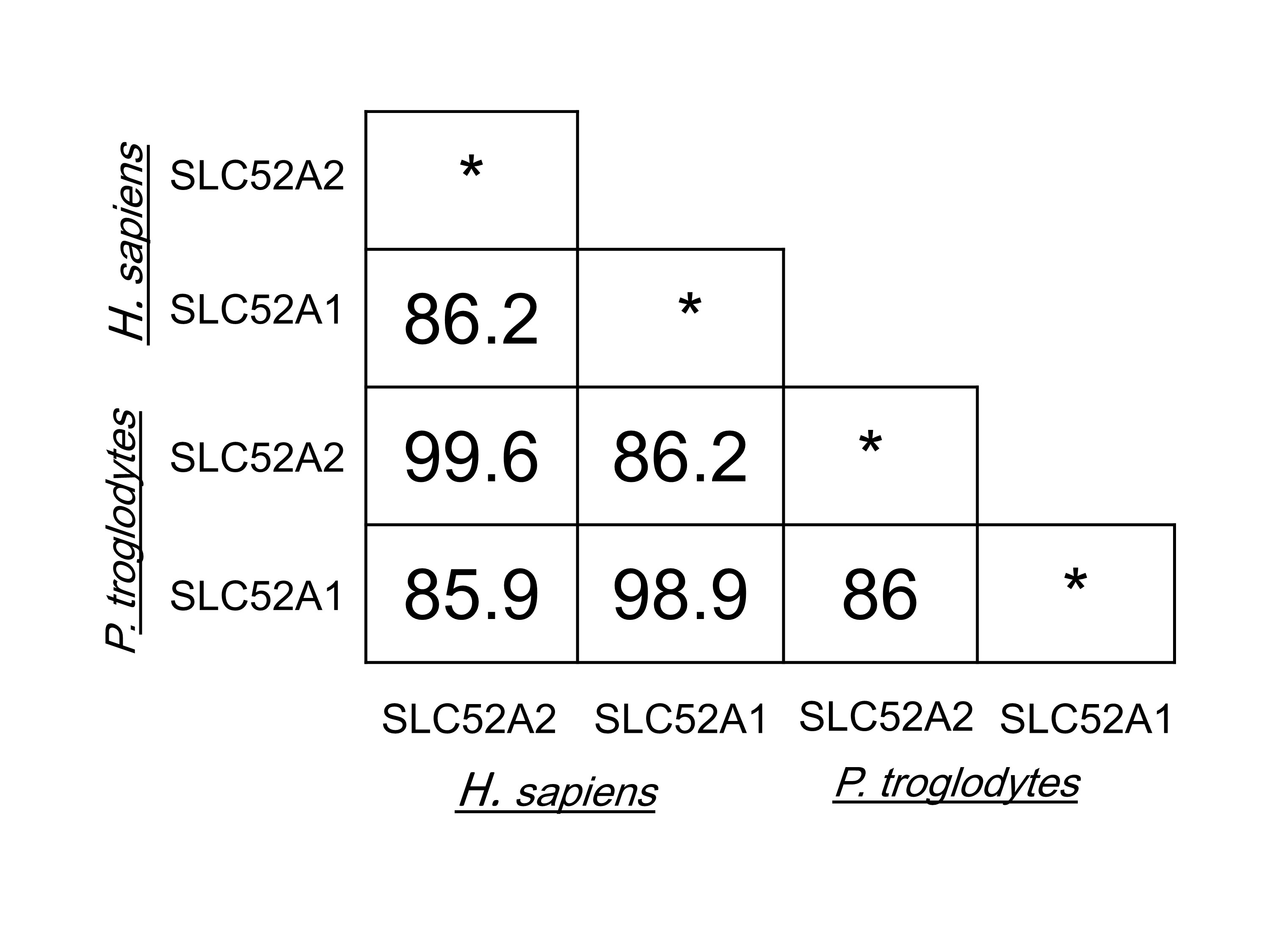

Supplement: FigureS1_veaf031 [file figures1_veaf031.jpeg]

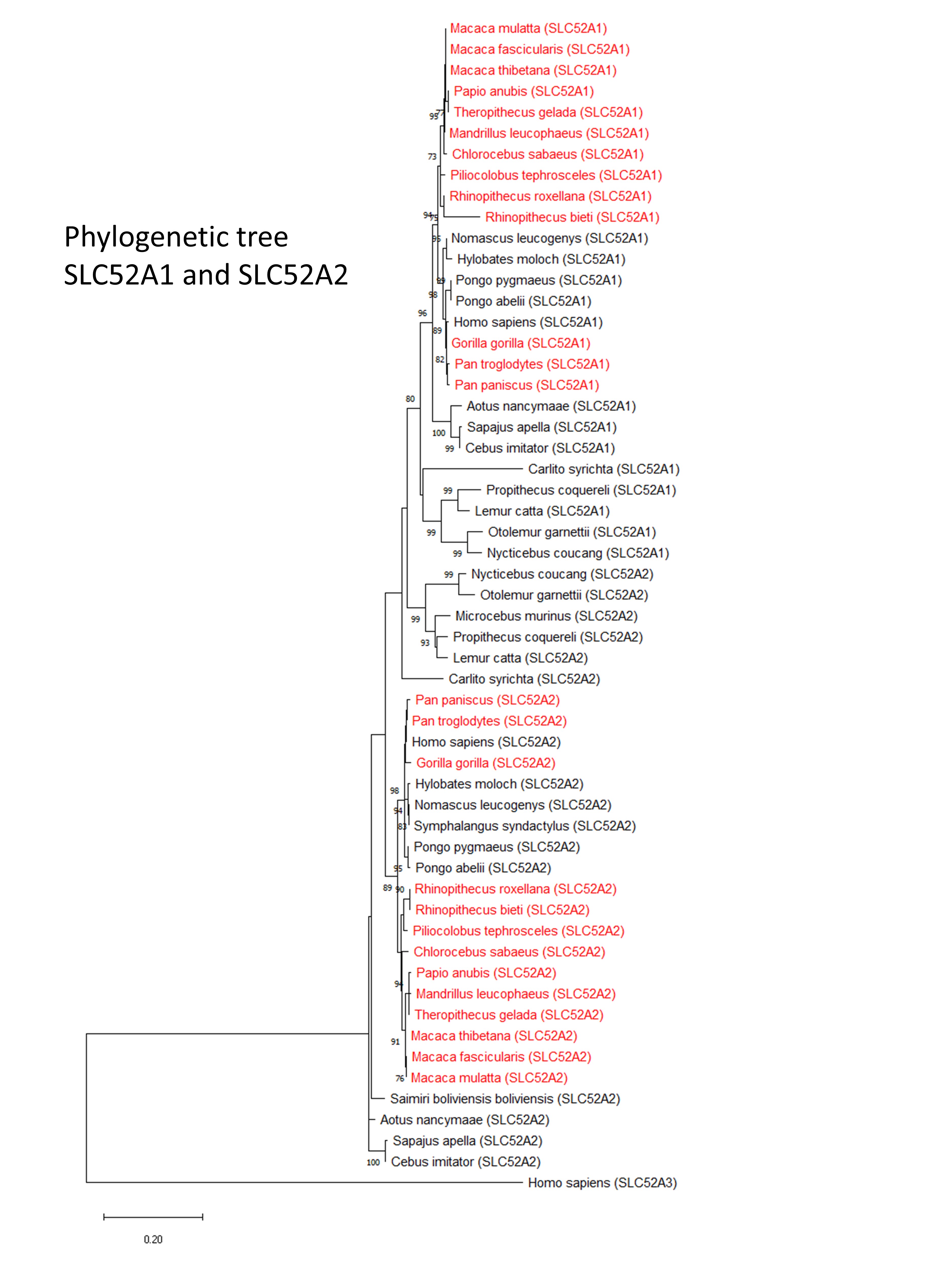

Supplement: FigureS2_veaf031 [file figures2_veaf031.jpeg]

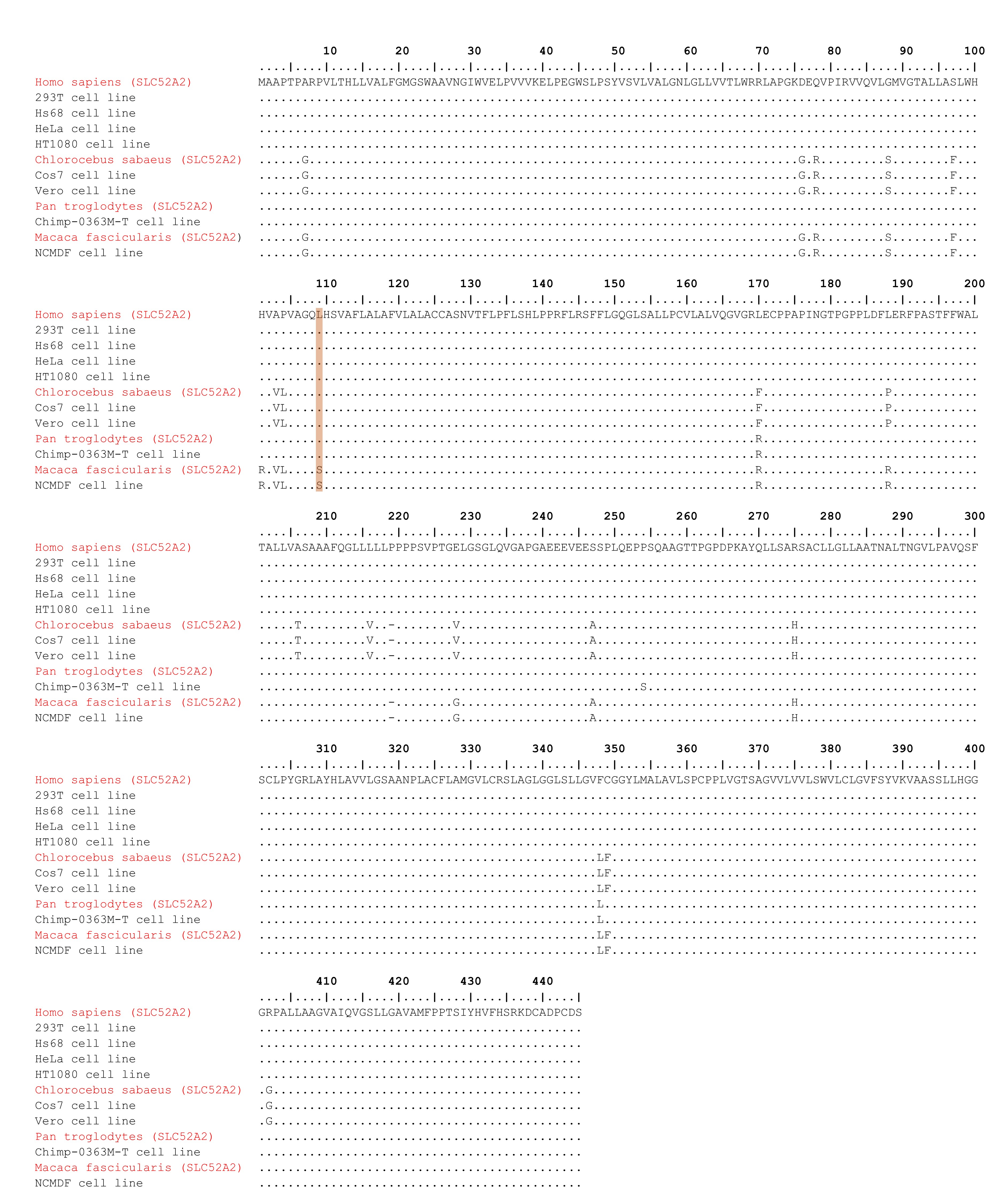

Supplement: FigureS3_veaf031 [file figures3_veaf031.jpeg]

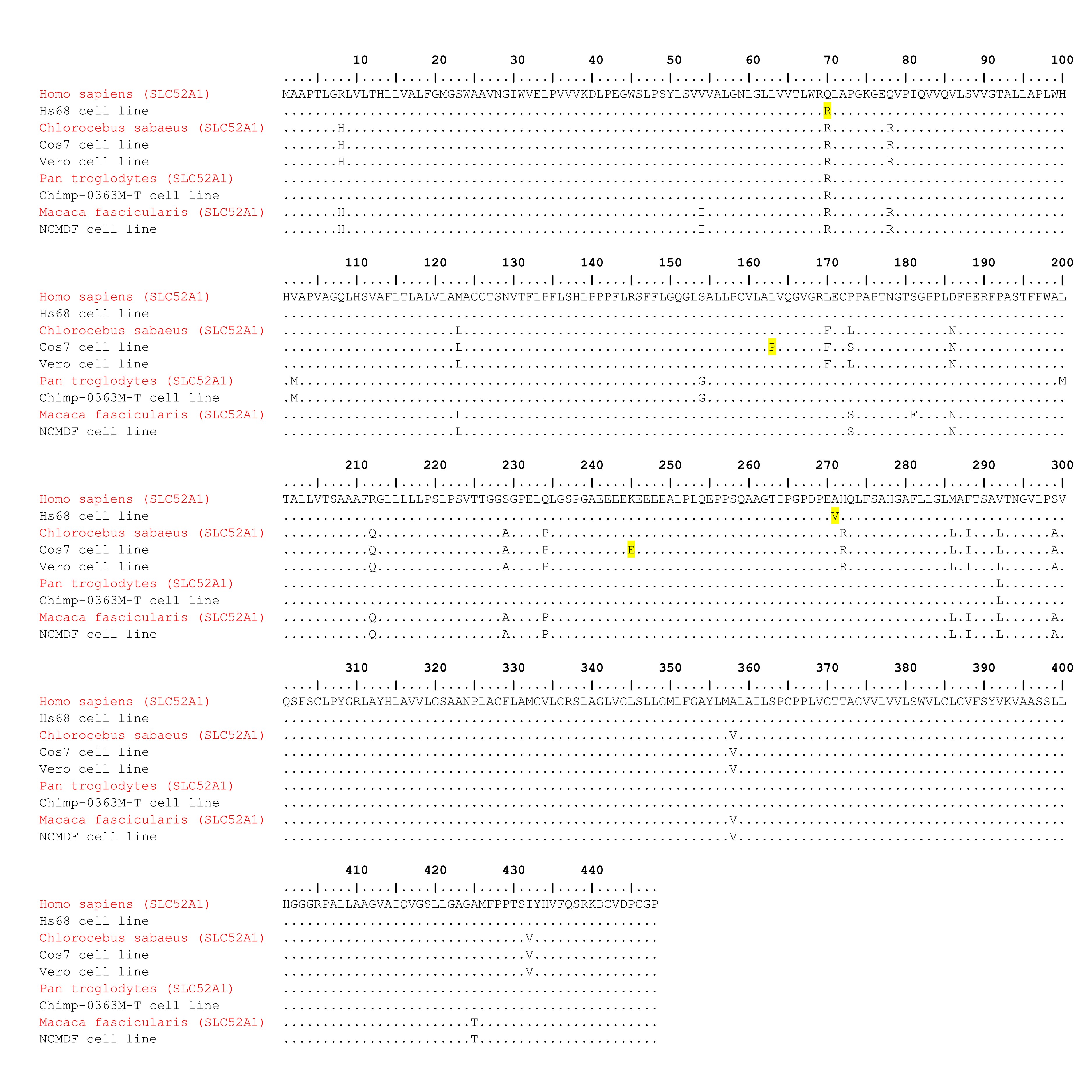

Supplement: FigureS4_veaf031 [file figures4_veaf031.jpeg]
